# Cell Cycle Re-entry in the Nervous System: From Polyploidy to Neurodegeneration

**DOI:** 10.3389/fcell.2021.698661

**Published:** 2021-06-24

**Authors:** Shyama Nandakumar, Emily Rozich, Laura Buttitta

**Affiliations:** Department of Molecular, Cellular and Developmental Biology, University of Michigan, Ann Arbor, MI, United States

**Keywords:** neurodegeneration, polyploidy, cell cycle, endomitosis, aging

## Abstract

Terminally differentiated cells of the nervous system have long been considered to be in a stable non-cycling state and are often considered to be permanently in G0. Exit from the cell cycle during development is often coincident with the differentiation of neurons, and is critical for neuronal function. But what happens in long lived postmitotic tissues that accumulate cell damage or suffer cell loss during aging? In other contexts, cells that are normally non-dividing or postmitotic can or re-enter the cell cycle and begin replicating their DNA to facilitate cellular growth in response to cell loss. This leads to a state called polyploidy, where cells contain multiple copies of the genome. A growing body of literature from several vertebrate and invertebrate model organisms has shown that polyploidy in the nervous system may be more common than previously appreciated and occurs under normal physiological conditions. Moreover, it has been found that neuronal polyploidization can play a protective role when cells are challenged with DNA damage or oxidative stress. By contrast, work over the last two and a half decades has discovered a link between cell-cycle reentry in neurons and several neurodegenerative conditions. In this context, neuronal cell cycle re-entry is widely considered to be aberrant and deleterious to neuronal health. In this review, we highlight historical and emerging reports of polyploidy in the nervous systems of various vertebrate and invertebrate organisms. We discuss the potential functions of polyploidization in the nervous system, particularly in the context of long-lived cells and age-associated polyploidization. Finally, we attempt to reconcile the seemingly disparate associations of neuronal polyploidy with both neurodegeneration and neuroprotection.

## Introduction

The prolonged maintenance of a non-dividing state is critical for the proper functioning of long lived cells in various tissues throughout the lifespan of an organism. The cells of the nervous system; neurons and glia, are some of the longest lived in many animals. It is known that maintaining a non-dividing state in these cells is critical for brain function (Frade, 2000; Aranda-Anzaldo and Dent, 2017).

In a majority of adult metazoan cells including neurons, muscles, and most epithelial cells, the G0 associated with terminal differentiation is thought to be permanent (Zacksenhaus et al., 1996; Cunningham et al., 2002; Huh et al., 2004; Buttitta and Edgar, 2007; O’Farrell, 2011). These cells exit the cell cycle with a diploid (2C) DNA content. Studies over the past few years have suggested that there are several overlapping and redundant biological pathways that influence the establishment and maintenance of G0 in terminally differentiated tissues, including the upregulation of the activity of negative regulators of the cell cycle, as well as changes in transcription and chromatin (Reviewed in Galderisi et al., 2003; Buttitta and Edgar, 2007; Oyama et al., 2014; Davis and Dyer, 2010; Duronio and Xiong, 2013; Ruijtenberg and van den Heuvel, 2016).

## Are All Terminally Differentiated Neurons Permanently Diploid, and in G0?

**FIGURE 1:**
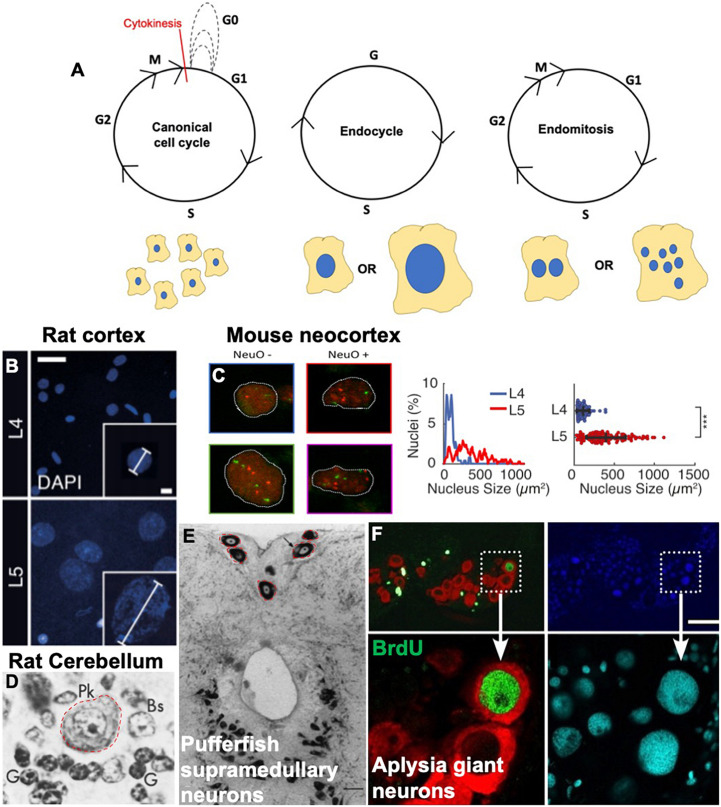
Variant cell cycles and polyploidy in neurons. **(A)** Cartoons showing the progression of the canonical cell cycle and two variant cell cycles: the endocycle and the endomitotic cycle. Multiple repeated canonical cell cycles result in numerous daughter cells with diploid DNA content, whereas endocycles result in cells with tetraploid or greater (>4C) DNA content and endomitosis can result in either binucleate or multinucleate cells. **(B–F)** Examples of polyploid neurons from the literature. **(B)** Nuclear DAPI staining and quantification showing larger, polyploid pyramidal neurons in the rat cortical layer https://doi.org/10.1016/j.celrep.2017.08.069. Scale bars = 25 and 5 μM for inset. **(C)** Polyploid neurons in the developing mouse neocortex from https://doi.org/10.1093/texcom/tgaa063. This study used a combination of flow cytometry and FISH combined with immunostaining against various neuronal markers to determine polyploidy. NeuO is a neuronal marker. Mouse neocortex has both polyploid neurons and non-neurons, both show increased number of red and green foci (FISH probes against loci on chromosomes 11 and 2, respectively). **(D)** Large polyploid purkinje neurons from rat cerebellum, outlined in red. Reprinted from Herman and Lapham (1973) with permission from Elsevier. License Number 5079560753665 (to author LB). **(E)** Red outlines and black arrows indicate polyploid supramedullary neurons of pufferfish Diodon holancthus stained with toluidine blue. Scale bar = 100 μM. Reprinted from Cuoghi and Marini (2001) with permission from Elsevier. License number 5053261503241 (to author SN). **(F)** Giant neurons in an Aplysia (slug) brain, positive for BrdU in green. Nuclei are stained with DAPI in blue and cyan, and red staining indicates FISH against mRNA of neurotransmitter achatin. Data from https://www.ncbi.nlm.nih.gov/pmc/articles/PMC6622835. Scale bar = 125 μM.

It has long been speculated that some neurons and glia in the CNS may be polyploid (Mann and Yates, 1973a; Yates and Mann, 1973; Bregnard et al., 1975). Recent work is beginning to confirm this, as well as indicate that polyploid cells in the nervous system may be more prevalent than previously thought, and these findings have important implications in the physiology and pathology of the nervous system. We begin with an introduction to variant cell cycles and examples of variant cell cycles in the nervous system across species (Figure 1).

### Variant Cell Cycles

The canonical cell cycle starts with a diploid cell containing two copies of each chromosome, and at the end of one cycle, results in two daughter cells, each diploid with two copies of each chromosome. Exceptions to this can be seen in several cell types and organisms across the animal and plant kingdoms (Edgar and Orr-Weaver, 2001; Frawley and Orr-Weaver, 2015). Variant cell cycles which give rise to a cell that contains more than two copies of the genome are classified as endoreduplication or endoreplication cycles. The resulting cell is polyploid in DNA content. There are different types of endoreplication cycles, and different contexts in which cells employ them to become polyploid.

Endoreplication cycles utilize parts of the cell cycle machinery to replicate DNA, but these cycles are curtailed and result in one cell with increased DNA content instead of two cells. Endoreplication cycles can involve only cycles of DNA replication and growth (termed endocycles) resulting in one nucleus with increased DNA content, or a cycle of with replication mitosis without an ensuing cytokinesis (termed endomitosis), resulting in two or more nuclei in one cell (Figure 1).

### Endocycles

Endocycles are variant cell cycles characterized by alternating Gap and DNA synthesis phases (Edgar and Orr-Weaver, 2001). In flies, endocycling is thought to be driven predominantly by an oscillation of Cyclin E/CDK2 activity and controlled by the transcriptional activity of E2F (Duronio and O’Farrell, 1995; Edgar and Orr-Weaver, 2001; Zielke et al., 2011; Moon and Kim, 2019). Another important factor that plays a role in endocycle progression is the APC/C^Frz/cdh1^ which ensures not only the degradation of mitotic CDKs, but also the timely degradation of geminin in S phase to prevent re-replication (Edgar et al., 2014). In mammals, variant or non-canonical E2Fs are employed specifically during endocycles implying a specialized role for these regulatory factors (Pandit et al., 2012; Matondo et al., 2018).

Several types of cells in various organisms employ endocycles during development or in contexts of cellular damage. Developmentally regulated endocycles occurs in some cells during development to aid the growth of the organism–cells generated by these endocycles usually possess several to several hundred copies of the genome, and often grow very large in size. It is interesting to note that developmentally regulated endocycles can generate cells of vastly varying ploidies depending on the tissue and context. While the enterocytes of the fly intestinal epithelium show average ploidies of 32–64C, nurse cells of the ovaries and cells of the salivary gland can be up to 1,024C (Frawley and Orr-Weaver, 2015).

Some examples of developmentally regulated endocycles in flies include the larval epidermis, salivary gland, fat body and some Sub-perineurial glia of the blood brain barrier (Hammond and Laird, 1985; Britton and Edgar, 1998; Lee et al., 2009; Unhavaithaya and Orr-Weaver, 2012; Von Stetina et al., 2018). In the adult fly, the enterocytes in the gut, the nurse and follicle cells of the ovary in adult females (Royzman et al., 2002; Fox and Duronio, 2013). These are all very large cells which either serve a biosynthetic demand or crucial barrier function. The cells resulting from these endocycles are usually constitutively polyploid.

While developmental endocycles have been well studied in *Drosophila*, they are also present and widespread in other eukaryotes. Several tissues in plants such as leaves, roots and trichomes have cells that endocycle after terminal differentiation to support growth (De Veylder et al., 2011; Frawley and Orr-Weaver, 2015; Lang and Schnittger, 2020). In mammals, the most studied example of endocycling is hepatocytes in the liver, and the trophoblast giant cells of the placenta. Just like in the fly, the different polyploid cells in mammals can exhibit varied levels of polyploidy. Polyploid hepatocytes contain 4–8C DNA content, however, trophoblast giant cells can have over 1,000 copies of the genome (Roszell et al., 1978; Severin et al., 1984; Zuckermann and Head, 1986; Jensen et al., 1989; Melchiorri et al., 1993; Zybina and Zybina, 1996; Klisch et al., 1999; Celton-Morizur and Desdouets, 2010). It is interesting to note here that highly polyploid cells such as nurse cells and trophoblast giant cells which provide critical trophic support are short lived, suggesting that the degree of polyploidy may influence the longevity of a cell. As cases of polyploidy continue to be uncovered, it is becoming clear this is a widely used cellular mechanism, yet the polyploid state remains poorly understood. Understanding the extent to which polyploidy is used during normal development and in abnormal conditions, will help reveal common features of the polyploid state. To facilitate communication across research areas, we have developed a searchable polyploidy literature atlas that encompasses organisms and model systems across eukarya. We envision this literature atlas could serve as a “living document,” an organizational structure and collection that will evolve as work on polyploidy progresses^1^.

In addition to cells that undergo developmentally regulated endocycles to become constitutively polyploid, some cells show a capacity to enter an endocycle in contexts of wounding and damage (facultative). These will be discussed in the following sections.

### Endomitosis

Endomitosis is another variant cell cycle which differs from endocycles in that it produces a cell with two or more nuclei. Endomitoses comprise a G_1_, S, G_2_, and a mitosis without cytokinesis (Edgar and Orr-Weaver, 2001; Frawley and Orr-Weaver, 2015). Thus, the regulation of endomitoses is different from that of an endocycle. Endomitotic cell cycles are characterized by a failure to undergo cytokinesis which results in binucleate or multinucleate cells. Endomitotic cells are less common than endocycling cells.

Endomitoses are best studied in the platelet-producing megakaryocyte cells in mammals (Zhang et al., 1996; Zimmet and Ravid, 2000; Ravid et al., 2002; Bluteau et al., 2009). Some SPGs in the fly blood brain barrier are known to become multinucleate by endomitosis (Eliades et al., 2010; Unhavaithaya and Orr-Weaver, 2012; Von Stetina et al., 2018). Examples of endomitosis giving rise to binucleate cells are cardiomyocytes in mouse and human hearts, lactating mammary epithelial cells and the binucleate cells of the *Drosophila* accessory gland (Stephen et al., 2009; Pandit et al., 2013; Paradis et al., 2014; Taniguchi et al., 2014, 2018; Box et al., 2019).

### Polyploidy in the Nervous System: From Mollusk to Man

#### Slugs do It Best

Sea slugs of the Aplysia species have long been used in studies of olfaction and memory formation (Coggeshall et al., 1970; Nagle et al., 1993; Sattelle and Buckingham, 2006; Moroz, 2011; Yamagishi et al., 2012, 2011; Kukushkin et al., 2019). These slugs possess giant neurons (roughly the size of one fly brain) which are perhaps the most extreme example of somatic polyploidy, possessing up to 600,000 copies of the genome (600,000C)! While we still do not know exactly why these neurons are so large, it is speculated that in “simpler” animals, one large cell can perform the functions of several smaller cells, trading off “complexity” for capacity (Frade and López-Sánchez, 2010; Mandrioli et al., 2010).

#### Drosophila

Endocycling has been observed in the *Drosophila* peripheral nervous system in the bristle cell lineage. Bristle cells are mechanoreceptive cells in the fly thorax. While it has been known for over 30 years that these cells become polyploid (up to 8C) during development (Fung et al., 2008), recent work has provided mechanistic insight into how these cells become polyploid. The bristle lineage consists of a neuron, a glial cell, a sheath cell, and one socket and one shaft cell. The shaft and socket cells become polyploid in a Cyclin A/CDK2 dependent manner, unlike most other tissues in fly which employ CyclinE/CDK2 oscillations to become polyploid (Audibert et al., 2005; Furman and Bukharina, 2008; Sallé et al., 2012).

The Sub-perineurial glia that form the protective blood brain barrier for the CNS in the fly become highly polyploid during development (Unhavaithaya and Orr-Weaver, 2012). These large cells adopt either an endocycle or an endomitosis depending on their location (Von Stetina et al., 2018) to become polyploid and support the rapidly growing larval brain during development. Inhibition of polyploidization in these cells results in impaired blood brain barrier function.

Our recent work has shown that neurons and glia become polyploid in the fly brain, specifically in the adult (Nandakumar et al., 2020). Our study found that the optic lobes show higher levels of polyploidy than the central brain and the ventral nerve cord. We also showed that an increase in polyploidy occurs within the first week after eclosion. In addition, exogenous DNA damage and oxidative stress can induce even higher levels of polyploidy, and the polyploid cells are protected from cell death.

#### Teleost Supramedullary Neurons

Several species of teleosts are also known to possess a small number of highly polyploid neurons called supramedullary neurons on the dorsal surface of the spinal cord or the rostral spinal cord (Nakajima et al., 1965; Bennett and Nakajima, 1967; Mola et al., 2001; Dampney et al., 2003). Depending on the species of fish, these neurons can have anywhere between 100 to over 5,000 copies of the genome. These neurons are very small in number, and have been proposed as a good *in vivo* model for electrophysiology studies due to their prominent size and convenient location. These large cells are thought to have a neuro-endocrine function as some species of puffer fish produce noradrenalin (Mola et al., 2002; Mola and Cuoghi, 2004). The need for biosynthesis of large amounts of adrenaline may underlie the polyploidy in these cells, however, this has not been functionally tested.

#### Other Vertebrates and Mammals

Initial observations of polyploidy in vertebrate brains involved the study of neurons and glia in the cerebellum by three different groups in the 1960s and 1970s (Lapham, 1963, 1968; Herman and Lapham, 1969, 1973; Lentz and Lapham, 1969, 1970; Lapham et al., 1971; Mann and Yates,1973a,b, 1979; Mann et al., 1976; Swartz and Bhatnagar, 1981). While these studies reported differing numbers, they concluded that the cerebellum does indeed possess polyploid cells. One study measured the proportion of polyploid cells at different ages in the human cerebellum and found that there was no increase in the proportion of polyploid of neurons or glia between ages 8 and 72, suggesting that unlike the liver and heart, the proportion of polyploidy remains constant in the human brain with age. These early studies speculated that the polyploidization may contribute to cerebellar memory and specialized function due to their increased transcriptional output (Mann and Yates, 1973b).

In addition to cytometric measurements, studies in the 1900s also made histological observations of neuronal nuclear hypertrophy in various mammals such as mice, rats, dogs, rhesus monkeys and even humans (Verhaart and Voogd, 1962; Lapham, 1963; Bregnard et al., 1975, 1979; Ribeiro, 2006; Toscano et al., 2009).

In mammals, most observations of neuronal polyploidy or hypertrophy report larger, mononucleate cells. However, there are a couple of very interesting exceptions: neurons of the dorsal root and pelvic ganglia, neurons of the superior cervical ganglion (SCG), neurons of enteric ganglia, and neurons innervating the heart (Bunge et al., 1967; Smith, 1970; Forsman et al., 1989; Ribeiro, 2006; Hunter et al., 2018). Binucleate SCG neurons have been observed in rats, rabbits, capybaras, guinea pigs, and humans.

The observations of larger, mononucleate polyploid neurons in the brain, and binucleate neurons in the autonomic nervous system also presents an interesting distinction worth exploring in future studies. What is the function of neuronal binucleation in involuntary actions? Does binucleation support secretory functions in neurons?

Modern genetic approaches investigating potential cell cycle re-entry in vertebrate brains began taking shape in the early 2000’s. In recent years, observations of bona fide polyploidy in neurons of the retinal ganglion of the chicken and mouse, cerebral cortex of the rat and neocortex of the mouse have been made using modern flow cytometry and high resolution imaging techniques (Morillo et al., 2010; López-Sánchez and Frade, 2013; Ovejero-Benito and Frade, 2015; Martin et al., 2017; Jungas et al., 2020). Work from the Frade lab has shown that the neurons of the retinal ganglion become tetraploid in an E2F-dependent manner. However, this endoreplication program is differentially regulated in chick and mouse central nervous system, as p27^kip1^ is necessary for tetraploidization in the chick, but not the mouse RGCs (López-Sánchez and Frade, 2013; Ovejero-Benito and Frade, 2013, 2015). Further advances in imaging and flow cytometry techniques have identified polyploid pyramidal neurons in the cerebrum of the rat, and the neocortex of the mouse, but the function and underlying cause for their polyploidy remain elusive (Sigl-Glöckner and Brecht, 2017; Jungas et al., 2020). These studies show sufficient evidence that polyploidization does indeed occur in higher vertebrates, suggesting that neuronal polyploidization may be a well conserved phenomenon. However, while these studies have made detailed observations of polyploidy in neurons, the precise function of polyploidization under each of these conditions remains unknown.

### Why Become Polyploid?

#### Increased Biosynthetic Capacity

Why do some cells become polyploid? What are the benefits of entering a variant cell cycle rather than undergoing cell division? Constitutively polyploid cells, as mentioned before, mainly perform two important functions: they usually have increased biosynthetic capacity, and they maintain barrier function (Reviewed in Edgar and Orr-Weaver, 2001; Lee et al., 2009; Øvrebø and Edgar, 2018). Polyploid cells with more copies of the genome can increase cell size and metabolic functions efficiently. Undergoing cell division involves cell rounding, cytoskeletal rearrangements and potential loss of cell-cell contacts (Sauer, 1935; Erenpreisa and Cragg, 2001; Lancaster et al., 2013; Frawley and Orr-Weaver, 2015). This can be problematic in cells performing important barrier functions. Endocycling can therefore be a way for these cells to grow in size and genome copy number without increasing in cell number.

Speculation about the role that tetraploidy plays in neurons has varied from generation of neuronal diversity to increased capacity for dendritic arborization. One study performed over 30 years ago (Szaro and Tompkins, 1987) compared the dendritic arbors of two *Xenopus* species, one diploid species and another which displays whole organism tetraploidy (where the entire organism has a larger genome). This study showed that while the brains from these two organisms were the same size, the neurons from the tetraploid species showed longer dendritic segments as well as larger dendritic arbors. This could mean that tetraploid neurons are able to make more synaptic connections and participate in larger neuronal networks, contributing to functional diversity. Polyploid neurons could also, as a virtue of increased biosynthetic capacity, increase production of neurotransmitters, resulting in robust signaling.

In glial cells, increased biosynthetic capacity in wrapping glia as a result of endocycling could ensure better sheathing of axon bundles and enhanced neuronal conductivity. Similarly, increased biosynthetic capacity could improve phagocytic glial function and aid in better clearance of cellular debris in the adult brain. Glial cells provide the bulk of the metabolic support to the neurons in the brain. In flies, glial glycolysis has been shown to be essential for neuronal survival (Volkenhoff et al., 2015) in the adult brain. Glial polyploidization might be a way for some glial cells in the central nervous system to increase their metabolic capacity.

#### Wound Healing and Compensatory Growth

Cells in the *Drosophila* adult abdominal epithelium respond to wounding by re-entering the cell cycle as well as undergoing cell fusion to become polyploid, and close the wound. Induction of the endocycle in these cells is dependent on the upregulation of E2F by the Hippo/Yorkie pathway as well as the degradation of mitotic cyclins by APC/C^Fzr^. Polyploidization is also known to play a role in wound healing in the mammalian corneal endothelium, heart and keratinocytes (Werner et al., 2007; Losick et al., 2013, 2016; Trakala and Malumbres, 2014; Trakala et al., 2015; Losick, 2016; Gandarillas et al., 2019; Grendler et al., 2019). Polyploid fat body cells of the *Drosophila* pupa and wax moth larvae respond to wounding by migrating to lesion sites and forming a “plug” to prevent infection by maintaining the epithelial barrier (Rowley and Ratcliffe, 1978; Franz et al., 2018).

Endoreplication has also been implicated in alternate modes of regeneration and response to cell loss. The liver remains best studied in this context as well in mammals, but recent studies have shown that polyploidization occurs in renal tubular epithelial cells in response to ischemic damage (Melchiorri et al., 1993; Lazzeri et al., 2018; Matsumoto et al., 2020). Other examples of endocycling in response to cell loss include the epicardium of the zebrafish heart (Uroz et al., 2019). In *Drosophila*, the enterocytes of the intestinal epithelium, the follicle cells of the ovary and the main cells of the accessory gland can cope with induced cell death by engaging a compensatory cellular hypertrophy or endocycle program to maintain tissue size and homeostasis (Tamori and Deng, 2013; Edgar et al., 2014; Shu et al., 2018; Øvrebø and Edgar, 2018; Box et al., 2019). In the fly optic lobe, where increase in polyploidy is accompanied by a steady loss of diploid cells, polyploidization may serve a compensatory role by enabling neurons to form more synaptic connections to compensate for cell loss to maintain visual acuity (Nandakumar et al., 2020).

#### DNA Damage Resistance and Repair

One additional benefit of polyploidy is resistance to DNA damage conferred by the number of copies of the genome–somatic mutations in one copy of a gene will not greatly impact the capacity of the cell to function since it will have many other copies of the genome. For over 80 years, scientists have observed that polyploid cells are able to endure and survive DNA damage better than diploid cells (Muntzing and Prakken, 1941). The resistance to DNA damage is attributed, in most part, to the number of copies of a gene that a polyploid cell has. If a cell has several “spares”, DNA damage caused by random somatic mutation to one copy of a crucial gene will not impede the cell’s ability to function or survive, as it will have more copies of the gene (D’Alessandro and d’Adda di Fagagna, 2017). The earliest studies on the resistance polyploid cells show to DNA damage were performed in the 1940s (Muntzing and Prakken, 1941). These studies compared the response of whole organism tetraploids to diploid rye plants and linked the resistance to radiation damage to ploidy variations.

Functional studies in genetic model organisms have since furthered our understanding of how some polyploid cells may resist DNA damage. The most prominent model used to understand the relationship between polyploidy and DNA damage resistance has been the various polyploid tissues in *Drosophila*. Studies in the follicle cells, fat body as well as salivary glands in the fly have shown that endocycling cells do not undergo apoptosis as a result of induced genome instability (Mehrotra et al., 2008). These polyploid cells can tolerate high levels of DNA damage, and harbor double strand breaks to their DNA, but do not undergo apoptosis. Further studies have shown that low levels of the tumor suppressor protein p53 in these endocycling cells is responsible for conferring their resistance to cell death (Mehrotra et al., 2008; Zhang et al., 2014). The tumor suppressor p53 is responsible for activating the expression of proapoptotic genes hid, reaper and grim in *Drosophila*, and these proteins are in turn upstream of the caspase cascade. Low levels of p53 in some *Drosophila* polyploid cells, combined with chromatin-level silencing of the pro-apoptotic genes confer high levels of resistance to DNA damage-induced cell death in these cells (Mehrotra et al., 2008; Zhang et al., 2014; Park et al., 2019).

Studies of cancer cells show that polyploidy can be induced by DNA damage. This is frequently observed in cancer cells which lack cell cycle checkpoints. Failure of cytokinesis or premature exit from the cell cycle without undergoing mitosis often results in tetraploid cancer cells. Several types of carcinomas with inactivated p53 or Rb have cells with hyperploid DNA content. Severe telomere attrition has been implicated in these cases as the source of DNA damage (Lazzerini Denchi et al., 2006; Davoli and de Lange, 2011). Polyploid cells are protected from DNA damage, and polyploidy can be induced by DNA damage. This suggests that polyploidy has been employed in several types of tissues and organisms as a robust adaptation to DNA damage.

Our recent work in *Drosophila* brains indicates that the rate of accumulation of polyploidy in adults can be exacerbated by damaging agents such as DNA damage or oxidative stress. Our experiments demonstrated that exogenous DNA damage leads to increased polyploidy, and that polyploid cells are protected from DNA damage induced cell death. Exposure to paraquat and UV both elicit a DNA damage response in the brain, and result in increased polyploidy.

Other work in *Drosophila* has shown that transposon silencing becomes compromised with age in the brain and has been linked with conditions of aging, neurodegeneration and decline in brain function (Abrusán, 2012; De Cecco et al., 2013; Li et al., 2013; Krug et al., 2017; Chang and Dubnau, 2019; Chang et al., 2019). This has been termed the “transposon storm” hypothesis of aging and neurodegeneration. Transposon reactivation has also recently been observed in aging fly guts, albeit at different levels (Riddiford et al., 2020). Could transposon reactivation represent a portion of the endogenous DNA damage that cells in the brain have to endure and overcome as they age?

Another potential source of DNA damage is DNA damage associated with high transcriptional activity (Hill et al., 2016; Langellotti et al., 2016). Highly transcribed loci in the genome are known to be susceptible to damage as a result of RNA:DNA hybrid formation. Recent work has shown that proteins implicated in neurodegenerative diseases such as TDP-43 are involved in preventing and contributing to repair at sites of transcription associated DNA damage. Age associated decline in TDP43 (Herrup and Arendt, 2002; Bonda et al.,2010a,b), coupled with high levels of transcription in neurons could contribute to unresolved DNA damage resulting from transcription-associated DNA lesions.

Transcriptional analysis of the aging fly brain shows age-associated reduction ATP metabolism, oxidative phosphorylation and cellular respiration (Nandakumar et al., 2020). This may indicate compromised mitochondrial function, which is a known hallmark of aging and a well known source of cellular oxidative stress (López-Otín et al., 2013). Compromised mitochondrial function can lead to increased levels of intracellular peroxide and superoxide radicals which can lead to oxidative DNA damage. Oxidized bases in DNA may evoke the need for base or nucleotide excision repair pathways to repair lesions.

## Conclusion and Future Perspectives

### Age-Dependent Accumulation of Polyploidy–A Common Theme in Long-Lived Tissues?

In the murine liver and the heart which have been extensively studied in the context of polyploidy: most cells are diploid at birth, with polyploidy appearing at the onset of weaning and acquisition of sexual maturity. A similar pattern of onset of polyploidization is also observed in the pancreas of mice and rats, the lacrimal glands of male rats. In addition, an increase in the proportion of polyploid cells with age has been observed and reported in the adrenal and thyroid glands (Teir, 1949; Geschwind et al., 1958; Carriere and Patterson, 1962; Paulini and Mohr, 1975; Gahan, 1977; Gilbert and Pfitzer, 1977; Roszell et al., 1978; Bohman et al., 1985; Nguyen and Ravid, 2010). In all of these cases, the proportion of polyploid cells increases rapidly at first, and then gradually over age.

The liver and lacrimal glands exhibit endocrine dependent onset of polyploidy, with the liver being dependent on thyroid and thymus function, and the lacrimal glands, on male gonads for polyploidization. The liver shows diet-dependent increase in polyploidy levels: rats on a restricted diet showed lower levels of accumulated polyploidy whereas rats feeding *ad libitum* showed higher levels of polyploidy accumulation with age, suggesting that the polyploidization of the liver is dependent on metabolic need and adaptive in nature (Paulini and Mohr, 1975; Enesco et al., 1991).

Similarly, observations of polyploidy and binucleation in cardiomyocytes have been made in several organisms (Brodsky et al., 1991, 1994; Hirose et al., 2019; Derks and Bergmann, 2020; Gan et al., 2020). Recent work has linked the onset of polyploidy to endocrine cues and show that the polyploidy is also marked by a metabolic shift from glycolysis to oxidative phosphorylation upon polyploidization (Hirose et al., 2019). Induced polyploidy in zebrafish hearts results in reduced regenerative capacity (González-Rosa et al., 2018). Further, binucleate cells and polyploidy increase with age as well as in diseased hearts (Clubb et al., 1987; Dzau and Gibbons, 1988; Lombardi et al., 1989; Brodsky et al., 1994; Derks and Bergmann, 2020). This has led to the prevailing notion that polyploidization in the heart is generally not beneficial. The current opinion in the cardiology field that binucleation directly hampers cardiac regeneration potential linking the lack of binucleation or polyploidization with regenerative capacity may be incomplete. Adult mammals and birds (endotherms) show cardiac polyploidy while amphibians and teleosts (ectotherms) do not (Derks and Bergmann, 2020). While most studies view polyploidization in the heart simply as a loss of regenerative potential, the idea that perhaps the acquisition of polyploidy, instead, is an adaptation to endothermic conditions and oxidative stress warrants further inquiry. Cardiomyocytes and neurons are among the longest lived cells in a mammalian body, perhaps polyploidization may underlie their longevity?

### Cell Cycle Re-entry and Neurodegeneration

A large body of work over the last two decades has drawn a link between cell cycle re-entry and neurodegeneration. The first studies describing this showed increased immunostaining for cell cycle proteins in conditions of neurodegeneration such as Alzheimer’s disease (AD) or AD models (Yang et al., 2006; Herrup and Yang, 2007; Khurana and Feany, 2007; Rimkus et al., 2008; Chen et al., 2010; Moh et al., 2011; Herrup, 2012; Frade and Ovejero-Benito, 2015). Since then, multiple models have been developed and several groups have corroborated this finding: brains exhibiting neurodegeneration also have cells that express cell cycle genes and proteins associated with the cell cycle. An enduring hypothesis emerged: that cell cycle re-entry in neurons is aberrant, and a marker of neurodegeneration including in human. Neurodegeneration is also marked by apoptosis and loss of neurons. The most common conclusion is that aberrant cell cycle re-entry causes cell death in neurons which, in turn, results in neurodegeneration. This hypothesis could also explain the appearance of neurons entering the cell cycle and bi-nucleate neurons even in pre-clinical cases of Alzheimer’s disease (Nagy, 1999, 2000; Zhu et al., 2008).

Markers of cell cycle re-entry have been observed in several other neuropathologies, including down’s syndrome (McShea et al., 1999), vascular dementia (Pelegrí et al., 2008), Huntington’s disease (Ranganathan and Bowser, 2003; Liu et al., 2015) and amyotrophic lateral sclerosis (ALS) (Ranganathan and Bowser, 2003; Liu et al., 2015; Manickam et al., 2018, 2020). In addition, neuronal cell cycle protein expression has been observed upon induction of iron toxicity (McShea et al., 1997; Wen et al., 2004), ischemia (Park et al., 2000; Marathe et al., 2015), and excitotoxicity (Chow et al., 2019; Iqbal et al., 2020). Altered metabolism and endocrine function have also been implicated in aberrant cell cycle re-entry in neurons (Atwood and Bowen, 2015). These studies suggest that cell cycle re-entry in the mammalian brain may be a common response to a plethora of acute as well as chronic neurological stressors.

**FIGURE 2:**
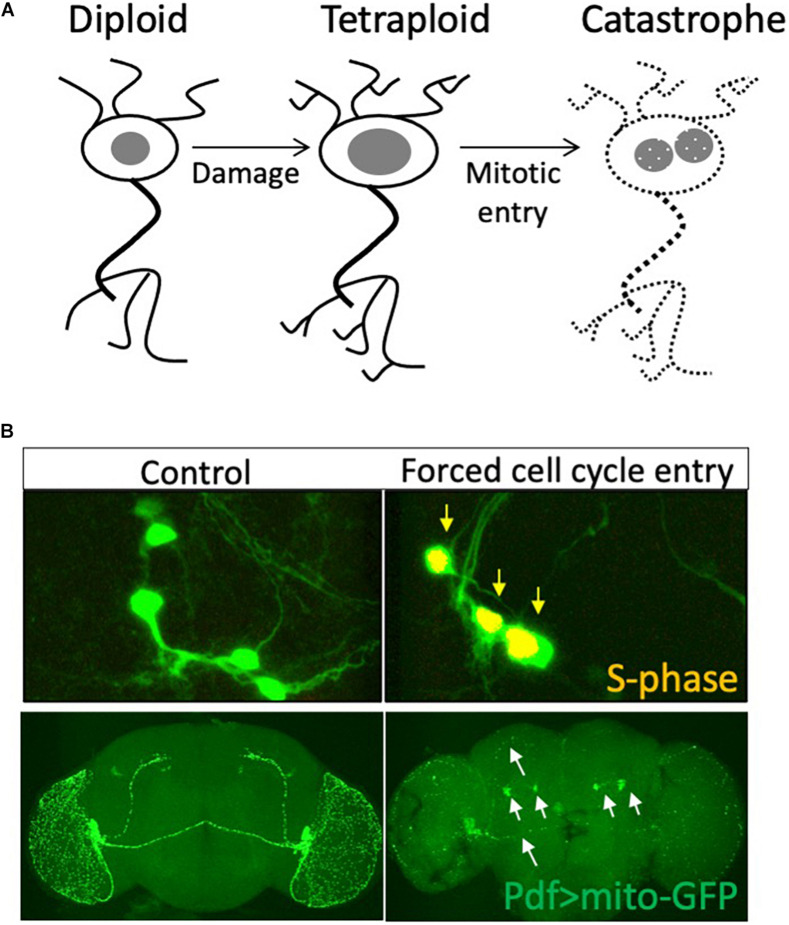
Is cell cycle re-entry both neuroprotective and neurodegenerative? **(A)** Neurons may enter the cell cycle and increase nuclear DNA content in response to tissue damage. This polyploid state may be neuroprotective, while further progression in the cell cycle into mitosis or sustained cell cycle re-entry may lead to axonal fragmentation and neurodegeneration. **(B)** We forced sustained cell cycle re-entry in postmitotic PDF neurons of the *Drosophila* brain and found this led to axonal fragmentation (white arrows) and degeneration of these neurons in the adult brain, abrogating circadian rhythm regulation. Data from Grushko and Buttitta (2015).

Is cell cycle re-entry associated with neurodegeneration always deleterious? Most studies published in the past two decades argue that cell cycle re-entry leads to cell death. However additional S-phase entry in differentiating neurons is not always associated with cell death (Ferguson et al., 2002; MacPherson et al., 2003). An alternate hypothesis is that cell cycle re-entry in neurons is not a cause, but rather a consequence of cell loss. Consistent with this hypothesis, our work in *Drosophila* has shown that neurons that undergo cell cycle re-entry and become polyploid are protected from cell death (Nandakumar et al., 2020), indicating that there are contexts where cell cycle re-entry in neurons is protective (Ippati et al., 2021). Congruent with our findings, a recent study using live imaging and fluorescent cell cycle reporters in the mouse hippocampus has shown that cell cycle entry in mature neurons protects cells from amyloid-beta toxicity and resultant cell death (Ippati et al., 2021). One possibility is that cell cycle re-entry that proceeds into mitosis leads to neurodegeneration (Ruggiero et al., 2012), while partial cell cycle re-entry is neuroprotective (Figure 2).

Currently the relationship between neurodegeneration-associated cell cycle re-entry and neuronal polyploidy remains unclear. Are these distinct phenomena? The inherent cellular diversity of the mammalian brain, the diversity of approaches and conditions used in the different studies cited make this a challenging question to address. Future studies using a combination of quantitative DNA content measurements, modern imaging techniques and genetically tractable model systems will shed light on the relationship between neuronal polyploidization and neurodegeneration.

## Author Contributions

SN and ER researched the literature and compiled the Polyploidy Atlas database. SN wrote the manuscript with input from ER and LB. All authors contributed to the article and approved the submitted version.

## Conflict of Interest

The authors declare that the research was conducted in the absence of any commercial or financial relationships that could be construed as a potential conflict of interest.

## Footnotes

1 https://sites.google.com/umich.edu/polyploidyatlas/home
